# Rotavirus NSP4 mediates an increase in the pro-inflammatory eicosanoid, prostaglandin E_2_, during infection

**DOI:** 10.1007/s00705-026-06782-3

**Published:** 2026-11-12

**Authors:** Willem J. Sander, Carolina H. Pohl, Sophia S. van der Schyff, Hester G. O’Neill

**Affiliations:** Department of Microbiology and Biochemistry, University of the Free State, PO Box 339, Bloemfontein, 9300 South Africa

**Keywords:** Rotavirus, PGE_2_, NSP4 viroporin, Ca^2+^ conductivity, Cytoplasmic phospholipase A_2_

## Abstract

Rotavirus (RV) type A is a leading cause of severe infantile gastroenteritis globally. Studies show that prostaglandin E_2_ (PGE_2_) levels increase during RV infection, enhancing viral attachment and internalisation. However, the underlying mechanism for the increased PGE_2_ levels remains unclear. Using a plasmid-based reverse genetics system, we engineered a recombinant RV carrying mutations in the viroporin domain of NSP4 that decreased cytoplasmic calcium (Ca^2+^) conductivity. This resulted in decreased cytoplasmic phospholipase A_2_ (cPLA_2_) activity, the rate-limiting enzyme in PGE_2_ biosynthesis, and subsequently reduced PGE_2_ levels and viral yield. These findings are consistent with RV-induced PGE_2_ production occurring in a NSP4-cyto[Ca²⁺]-cPLA_2_-dependent manner.

**Supplementary Information:**

The online version contains supplementary material available at https://doi.org/10.1007/s00705-026-06782-3.

## Introduction

Rotavirus (RV) is a leading cause of severe gastroenteritis in infants and children under the age of five [1]. The virus has a triple-layered virion containing 11 double-stranded RNA genome segments, encoding six structural (VP1–VP4, VP6, and VP7) and five to six non-structural proteins (NSP1–NSP6) [2]. During replication, NSP4 plays a central role in viral morphogenesis, facilitating translocation of double-layered particles to the endoplasmic reticulum (ER) for outer capsid acquisition [2]. In addition, secreted NSP4 functions as an enterotoxin, playing a crucial role in RV pathogenesis. This form can modulate cytoplasmic Ca^2+^ (cyto[Ca^2+^]) in uninfected cells, resulting in watery diarrhoea. Furthermore, during replication, NSP4, via its viroporin domain, also causes Ca^2+^ to leak from stores within the ER, enhancing viral autophagy, nucleation of RV viroplasms, and assembly of the outer capsid protein, VP7 [3–7].

RV infection increases production of the immunomodulatory eicosanoid, prostaglandin E_2_ (PGE_2_), in MA104 cells, which enhances viral attachment and internalisation [8]. This supports previous work suggesting that PGE_2_ exerts a proviral effect during the early stages of RV replication [9]. Cytoplasmic Ca^2+^ plays an important role in PGE_2_ biosynthesis [10] since it is crucial for the activation of cytoplasmic phospholipase A_2_ (cPLA_2_), the rate-limiting enzyme in PGE_2_ biosynthesis [11]. In the current study we investigated if the NSP4-mediated increase in cyto[Ca^2+^] drives PGE_2_ production, resulting in a proviral environment, using a plasmid-only RV reverse genetics system [12].

## Materials and methods

HEK293, MA104 and BSR-T7/5 cells were maintained in Dulbecco’s modified Eagle medium (DMEM; Gibco) supplemented with 5% foetal bovine serum (Gibco), 1% Penicillin-Streptomycin-Amphotericin B (Gibco), and 1% nonessential amino acids (Gibco) at 37 °C and 5% CO_2_. Rotavirus SA11 [13] was propagated in MA104 cells and viral yield determined using 50% tissue culture infectious doses (TCID_50_) [14]. Pancreatic porcine trypsin type IX (1 µg/ml; Sigma Aldrich) was added during all viral replication experiments.RV plasmids pT7-VP1SA11 through pT7-NSP6SA11 (Addgene) [12] were used to rescue recombinant and mutant viruses. A mutated genome segment, introducing mutations in the viroporin domain of NSP4 (GenScript), was used to generate the mutant plasmid using the NEBuilder^®^ HiFi DNA Assembly Cloning Kit (New England Biolabs; Supplementary data).

To rescue recombinant SA11 (rSA11), BSR-T7/5 cells were co-transfected with the 11 transcription plasmids and helper plasmids using TransIT-LT1 (Mirus Bio) [12]. Mutant virus rSA11-mNSP4 was rescued by replacing pT7-NSP4SA11 with pT7-mNSP4SA11. After 48 h, MA104 cells were added to transfected BSR-T7/5 cells in the presence of trypsin. After three days, co-cultured cells were freeze-thawed three times and passaged on MA104 cells. Rescue was confirmed by RT-PCR, restriction enzyme digestion targeting NSP4 (XbaI) and NSP2 (BstUI), and whole genome sequencing (Supplementary data) [15].

To quantify PGE_2_, HEK293 cells were infected at multiplicity of infection (MOI) 5 with rSA11 or rSA11-mNSP4 and PGE_2_ measured at 2 h and 6 h post infection. Virus was adsorbed to cells for 1 h at 37 °C, following which the inoculum was removed, cells were washed, and fresh medium was added for the remainder of the infection period. The 2 and 6 h time points were measured from the point of inoculum addition. Prostaglandins were extracted using C18 solid phase extraction columns and quantified by enzyme-linked immunosorbent assay (ELISA; Cayman Chemicals) [8]. To prevent lipid peroxidation, citric acid (80 mM) and butylated hydroxytoluene (0.1% v/v) were added prior to extraction.

Cytoplasmic Ca^2+^ was measured using pinocytic loading of Fura-dextran into HEK293 cells as described before [16], with modifications. Briefly, 1 × 10^4^ HEK293 cells were seeded into 96-well plates and infected at MOI 20 with rSA11 or rSA11-mNSP4 for 1 h. Ca²⁺ measuring via Fura-dextran relies on detecting a transient, population-averaged fluorescence signal within a short acquisition window, and a higher fraction of infected cells (achieved via higher MOI) was required to obtain a reliably detectable signal above assay noise. Lipopolysaccharide (LPS) was used as a positive control for increased intracellular Ca^2+^, and 1,2-bis-(2-aminophenoxy)ethane-N, N,N’,N’-tetraacetic acid (BAPTA-AM; 10 µM) was included as a Ca^2+^ chelator control. Fluorescence readings were taken every 15 min at alternate excitation wavelengths (340/380 nm) with emission filtered at 530 nm.

cPLA_2_ activity was measured using a cPLA_2_ assay kit (Cayman Chemical) in HEK293 cells infected (MOI = 5) with rSA11 or rSA11-mNSP4 at 2 h and 6 h post infection, in the presence or absence of BAPTA-AM. Samples were pre-treated with iPLA_2_- and sPLA_2_-specific inhibitors to ensure selective measurement of cPLA_2_ activity, expressed as nmol/min/mL. Viral RNA yield was determined by RT-qPCR targeting genome segment 6 (GS6) at 2 h and 6 h post infection as described previously [8].

All assays were performed in at least triplicate biological replicates on separate days, with each repeat analysed in triplicate (*n* = 3). Data are presented as means ± standard error of the mean. Statistical analysis was performed using two-way ANOVA with a Tukey-Kramer test in R Studio. A p-value of < 0.05 was considered statistically significant.

## Results

**Fig. 1:**
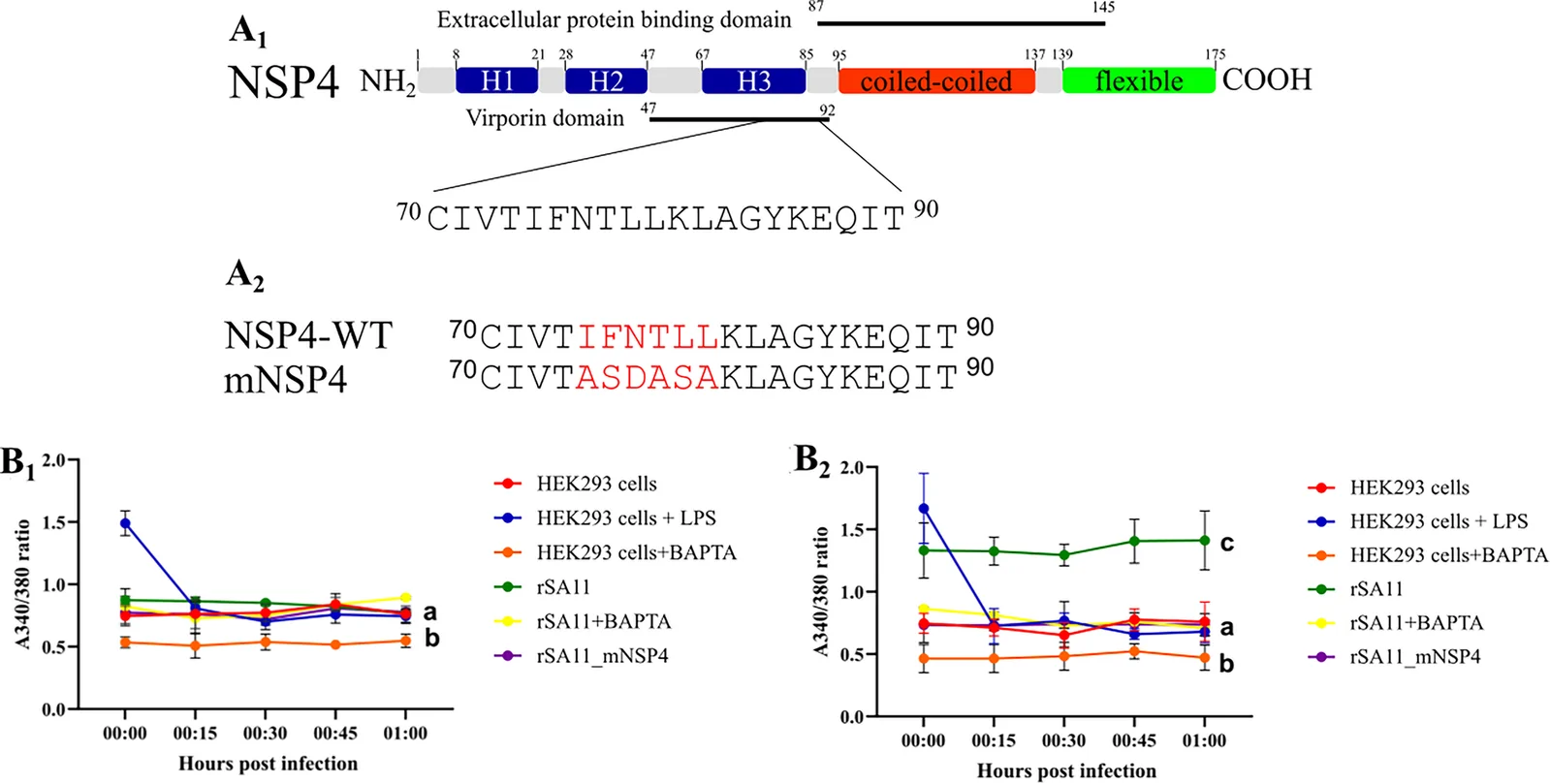
Engineering of rSA11-mNSP4 mutant rotavirus. A1) Schematic representation of NSP4 and primary sequence of the viroporin region (aa70–90). A2) Alignment of the NSP4 proteins corresponding to wild-type (NSP4-WT) and mutated (mNSP4) sequences. The activity of Ca^2+^ during rSA11 + BAPTA and rSA11-mNSP4 infection (MOI = 20) as determined by Fura-dextran was compared to rSA11-infected HEK293 cells at B1) 2 h and B2) 6 h post infection. Error bars indicate the standard error of the mean (*n* = 3). Lowercase letters denote significant differences (*p* = 0.004; rSA11 + BAPTA) for cytosolic Ca^2+^ at the 2 h time point and significant differences (*p* = 0.015, rSA11 + BAPTA; *p* = 0,0001, rSA11) for cytosolic Ca^2+^ at the 6 h time point

To investigate the mechanism driving RV-induced PGE_2_ production, a recombinant RV carrying mutations in the viroporin domain of NSP4 (rSA11-mNSP4) was engineered to cause defective Ca^2+^ conductivity [17]. The rescued rSA11-mNSP4 was confirmed to carry the mutations introduced in the viroporin domain (aa 75–80) of NSP4 (Fig. 1A; Supplementary data). It is important to note that Illumina MiSeq sequencing of the rescued virus also indicated additional mutations in the enterotoxin domain: T134A, M135A and T137S. Since it was previously shown that the enterotoxin domain has no effect on increasing cyto[Ca^2+^] [18], we continued with the rescued virus.To more closely reflect the human in vivo responses, the effect of rSA11-mNSP4 on cytosolic Ca^2+^ concentrations was determined in the human embryonic kidney cell line, HEK293, using Fura-dextran. The addition of lipopolysaccharide immediately prior to reading the assay, increased the A340/A380 ratio, confirming successful assay establishment. When comparing Ca^2+^ concentrations at 6 h post infection, cells infected with rSA11 in the presence of BAPTA showed a significant decrease compared to cells infected with rSA11 only (Fig. 1B). Importantly, a comparable decrease was observed in cells infected with rSA11-mNSP4, confirming that the mutations introduced into the viroporin domain successfully reduced Ca^2+^ conductivity (Fig. 1B).

**Fig. 2:**
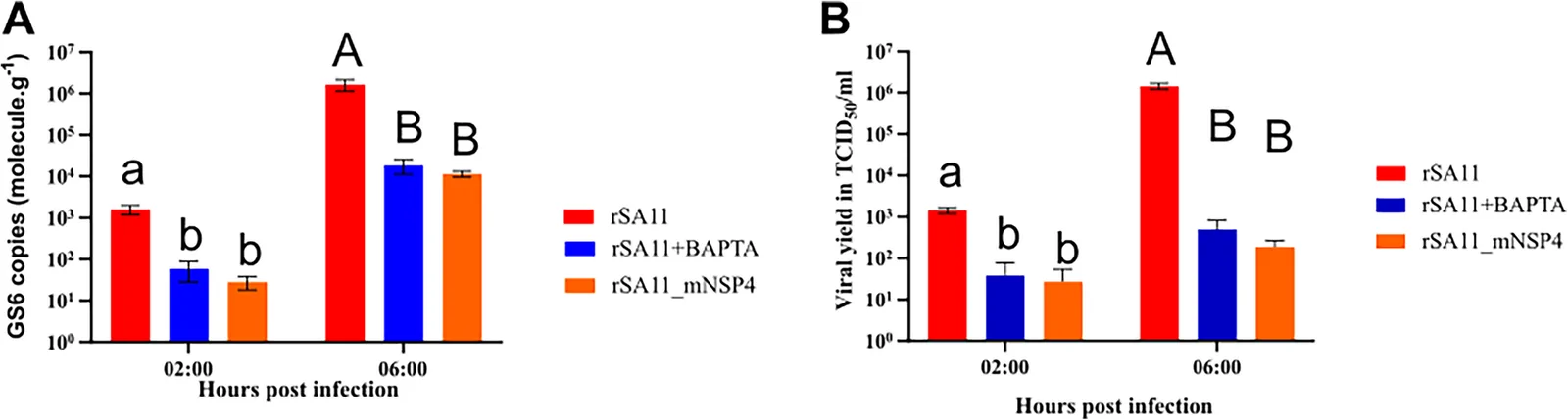
Effect of NSP4 mutation on rotavirus replication. (**A**) Copy number of GS6 as determined by RT-qPCR and comparative rotavirus yield as determined by TCID_50_ (**B**) at 2 h and 6 h post infection for rSA11-mNSP4 compared to rSA11-infected HEK293 cells. Lowercase letters denote significant differences [(*p* = 0.0015, rSA11 + BAPTA; *p* = 0.0014, rSA11_mNSP4) for GS6 copies; (*p* = 0.0003, rSA11 + BAPTA; *p* = 0.00034, rSA11_mNSP4) for viral yield] among groups at the 2 h time point, and uppercase letters denote significant differences [(*p* = 0.0026, rSA11 + BAPTA; *p* = 0.0025, rSA11_mNSP4) for GS6 copies; (*p* = 0.00031, rSA11 + BAPTA; *p* = 0.00031, rSA11_mNSP4) for viral yield] among groups at the 6 h time point

To determine the influence of reduced Ca^2+^ conductivity on viral replication, infectious viral yield was evaluated at 2 h and 6 h post infection. Infection with rSA11-mNSP4 for 6 h resulted in a decrease in both viral yield and GS6 copy number compared to rSA11 (Fig. 2A, B). The magnitude of this decrease was comparable to that observed in rSA11-infected cells treated with BAPTA at both time points (Fig. 2A, B).

**Fig. 3:**
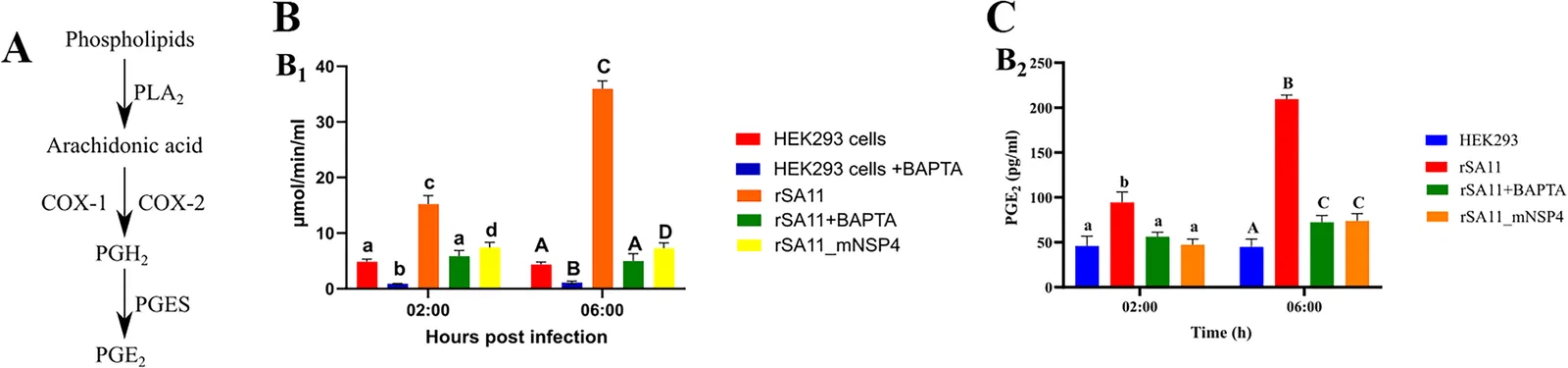
PGE_2_ biosynthesis and production. **A**) Simplified PGE_2_ biosynthetic pathway showing the release of arachidonic acid (AA) from phospholipids by cytoplasmic phospholipase A_2_ (cPLA_2_). Arachidonic acid is subsequently converted to PGH_2_ by cyclooxygenases (COX-1/2) which, in turn, is converted to PGE_2_ by prostaglandin E synthetases (PGES). **B**) The activity of cytoplasmic phospholipase A_2_ during rSA11 + BAPTA and rSA11-mNSP4 infection as determined by phospholipase A_2_ assay compared to rSA11-infected HEK293 cells at 2 h and 6 h post infection. C) The amount of PGE_2_ produced during rSA11 + BAPTA and rSA11-mNSP4 infection as determined by ELISA compared to rSA11-infected HEK293 cells at 2 h and 6 h post infection. Error bars indicate the standard error of the mean (*n* = 3). Lowercase letters denote significant differences [(*p* = 0.0008, rSA11 + BAPTA; *p* = 0.0005, rSA11_mNSP4) compared to rSA11 for cPLA_2_ activity; (*p* = 0.0003, rSA11 + BAPTA; *p* = 0.0003, rSA11_mNSP4) compared to rSA11 for PGE_2_ production] among groups at the 2 h time point, and uppercase letters denote significant differences [(*p* = 0.000004, rSA11 + BAPTA; *p* = 0.000005, rSA11_mNSP4) compared to rSA11 for cPLA_2_ activity; (*p* = 0.0004, rSA11 + BAPTA; *p* = 0.004, rSA11_mNSP4) compared to rSA11 for PGE_2_ production] among groups at the 6 h time point

Increased cyto[Ca^2+^] is crucial for the activation and translocation of cPLA_2_, the rate-limiting enzyme in PGE_2_ biosynthesis, from the ER to stores of arachidonic acid (Fig. 3A). To investigate the effect of the reduced cyto[Ca^2+^] on cPLA_2_ activity and a possible mechanistic link between NSP4-mediated Ca^2+^ conductivity and PGE_2_ biosynthesis, cPLA_2_ activity was measured in the presence of BAPTA and in rSA11-mNSP4-infected cells at 2 h and 6 h post infection. At both time points, a significant decrease in cPLA_2_ activity was observed for rSA11 + BAPTA and rSA11-mNSP4 compared to rSA11 (Fig. 3B). The mutations in NSP4 also resulted in decreased PGE_2_ production in HEK293 cells at both 2 h and 6 h post infection (Fig. 3C) and was comparable to that seen in rSA11-infected cells treated with BAPTA (Fig. 3C), emphasising the importance of Ca^2+^ in PGE_2_ production. Taken together, these results indicate that the mutations introduced into the viroporin domain of NSP4 reduced cyto[Ca^2+^], subsequently limiting cPLA_2_ activity, decreasing PGE_2_ production, and reducing both viral progeny and RNA yield.

## Discussion

RV infection increases production of PGE_2_ in MA104 cells, and this increase enhances viral attachment and internalisation [8]. This supports work [9] suggesting that PGE_2_ exerts its proviral effect during the early stages of RV replication and that, depending on the timing of treatment with genipin, PGE_2_ inhibition affects either early stage (attachment and internalisation) or late stage (assembly and egress) viral replication [19]. It is well established that NSP4, via its viroporin domain, causes Ca^2+^ to leak from the ER, which is critical for several steps in RV replication including viral autophagy, nucleation of viroplasms, and outer capsid assembly [3–5]. Increased cyto[Ca^2+^] is furthermore crucial for the activation and translocation of cPLA_2_ from the ER to stores of arachidonic acid, the rate-limiting step in PGE_2_ biosynthesis [20].

In this study we show that an NSP4-mediated increase in cytoplasmic Ca²⁺ is associated, and consistent with driving cPLA_2_ activation, leading to increased PGE_2_ synthesis during RV infection. Treatment of RV-infected cells with BAPTA, a chelator of cyto[Ca^2+^], significantly decreased cPLA_2_ activity and consequently reduced PGE_2_ production. A subsequent decrease in viral progeny and RNA yield was observed, concurring with previous studies [3, 21]. Furthermore, mutations in the viroporin domain of NSP4 which reduced Ca^2+^ conductivity [17], produced comparable decreases in cPLA_2_ activity, PGE_2_ production, viral progeny, and RNA yield, directly implicating NSP4 as the viral mediator of this pathway. It must be noted that the difference in MOI at which experiments were performed limits direct quantitative comparison of the absolute signal magnitude between the Ca²⁺ dataset and the PGE_2_/cPLA_2_ datasets, although within-assay analyses were performed at a consistent MOI for each assay.

While previous work reported an inability to rescue NSP4 viroporin mutants [6]—highlighting the essential nature of this domain for viral viability and Ca^2+^-dependent signalling—we were able to recover a viable mutant virus, albeit with markedly reduced replication efficiency, reaching maximal titres below 10² TCID_50_/ml at 6 h post infection. This attenuation is consistent with impaired viroporin-mediated Ca²⁺ flux and further supports the essential role of NSP4 in regulating Ca^2+^-dependent replication processes. Although the rescued virus had additional amino acids changes at T134A, M135A and T137S in the enterotoxin domain, which does not affect cytosolic Ca^2+^ levels [18], the effect of the observed changes on viral viability cannot be ruled out. We did not directly verify mutant NSP4 expression or stability by Western blot analysis; while the successful rescue and continued viability of rSA11-mNSP4 argue against gross protein destabilisation, we cannot exclude a contribution of reduced NSP4 protein production or misfolding to the phenotypes observed, which is a limitation of the study.

The observation that cPLA_2_ activity in uninfected HEK293 cells remained lower than that observed in rSA11-mNSP4-infected cells suggests the existence of a secondary, Ca^2+^-independent mechanism, contributing to cyto[Ca^2+^] elevation during infection. We postulate that this could be due to the additional regulation of cPLA_2_ by MAP kinase [22]. It is therefore possible that NSP2, which has kinase-like activity [23, 24] phosphorylates cPLA_2_ early during infection, but this hypothesis needs to be further investigated.

The increased PGE_2_ production resulting from NSP4-driven Ca^2+^ elevation likely creates a proviral environment, possibly mediated through enhanced viral internalisation, resulting in increased viral yield [8]. The data support a model in which increased levels of the pro-inflammatory eicosanoid PGE_2_ during RV infection are induced in a NSP4-cyto[Ca²⁺]-cPLA_2_-dependent manner, although formal epistasis between NSP4-mediated Ca²⁺ elevation and cPLA_2_/PGE_2_ induction remain to be directly tested.

## Supplementary Information

Below is the link to the electronic supplementary material.

Supplementary Material 1

## Data Availability

All data supporting the findings of this study are available within the paper and its Supplementary Information.
