# Supplementary material for: Rotavirus NSP4 mediates an increase in the pro-inflammatory eicosanoid, prostaglandin E2, during infection
Source: Arch Virol. 2026 Nov 12;171(12):320. doi: 10.1007/s00705-026-06782-3 (PMC13759588; doi:10.1007/s00705-026-06782-3)
Supplement: Supplementary file 1 [file 705_2026_6782_MOESM1_ESM.docx]

**Supplementary Methods**

**Plasmid construction**

RV plasmids pT7-VP1SA11, pT7-VP2SA11, pT7-VP3SA11, pT7-VP4SA11, pT7-VP6SA11, pT7-VP7SA11, pT7-NSP1SA11, pT7-NSP2SA11, pT7-NSP3SA11, pT7-NSP4SA11 and pT7-NSP5SA11 (Addgene) [12] were used to rescue recombinant and mutant viruses with some modifications. Silent mutations, identified with WatCut online tool (<http://watcut.uwaterloo.ca/>), were introduced in pT7-NSP2SA11 (BstUI; pT7-sNSP2SA11) and pT7-NSP4SA11 (XbaI; pT7-sNSP4SA11) using site-directed mutagenesis (Supplementary Table 1).

**Rescue of mutant RVs**

BSR T7/5 cells were seeded in 6-well plates (7×10^5^ cells per well) and incubated for 24 hours at 37°C and 5 % CO_2_. At 90 % confluency, the cells were rinsed and serum-free DMEM was added to the cells. To rescue recombinant SA11 (rSA11), the 11 transcription plasmids encoding the individual RV genome segments (2250 ng for pT7-sNSP2SA11 and pT7-NSP5SA11; 750 ng for the remaining plasmids) were co-transfected with helper plasmids as described before [12]. To rescue rSA11-mNSP4, pT7-sNSP4SA11 was replaced with pT7-mNSP4SA11. Transfections were performed with 30 μL of TransIT-LT1 transfection reagent (Mirus Bio, USA). The cells were incubated with the transfection mix for 24 h DMEM at 37°C and 5 % CO_2_. Forty-eight hours later, 5×10^5^ MA104 cells were added to the transfected BSR T7/5 cells, and pancreatic porcine trypsin type IX was added to a final concentration of 1 μg/mL. After three days at 37°C and 5 % CO_2_, the co-cultured cells were frozen and thawed three times before passaging on MA104 cells.

To confirm rescue of rSA11 and the mutant viruses, RNA was extracted using TRIzol (Invitrogen, USA) as described before [16], followed by RT-PCR (Supplementary Table 1) using AMV First Strand cDNA Synthesis (Promega, USA) according to the manufacturer’s instructions. Partial coding sequences for NSP2 and NSP5 were subsequently amplified using Phusion DNA polymerase (Thermo Fischer Scientific) and the following cycling parameters: 95°C for 2 min; 25 cycles of 98°C for 10 s, 55°C for 15 s and 72°C for 30 s. Purified amplicons were then subjected to restriction enzyme digestions targeting genome segments encoding NSP2 (BstUI; Thermo Fischer Scientific, USA) and NSP4 (XbaI; Thermo Fischer Scientific, USA). The whole genome sequences of the viruses were subsequently determined [16].

**Supplementary Table 1 – Primers used in the study**

| **Primer Name** | **Sequence (5’ → 3’)** | **Features** | **Purpose** |
| --- | --- | --- | --- |
| MUTNSP4_F | ATTGCACACAAT**T**CT**A**GAGGATCCAGGA^*#^ | XbaI | Site-directed mutagenesis |
| MUTNSP4_R | TCCTGGATCCTC**T**AGAATTGTGTGCAAT | XbaI | Site-directed mutagenesis |
| MUTNSP2_F | AGCTATGCTGAC**C**GC**G**AAAGTAGACAAA | BstUI | Site-directed mutagenesis |
| MUTNSP2_R | TTTGTCTACTTT**C**GC**G**GTCAGCATAGCT | BstUI | Site-directed mutagenesis |
| pT7mNSP4_F | AATGTTCATATAAAGTGG | - | Construction of pT7-mNSP4SA11 |
| pT7mNSP4_R | AGGTCCGATGGTGCTTAC | - | Construction of pT7-mNSP4SA11 |
| mNSP4_F | GTAAGCACCATCGGAACCTGATGGCTGACTGAG | - | Construction of pT7-mNSP4SA11 |
| mNSP4_R | CCACTTTATATGAACCATTTTGACGTTTTCAATGCAATTTTC | - | Construction of pT7-mNSP4SA11 |
| PCR_NSP2_F | GGTCACATAAGCGCTTTCTA | - | Amplification of NSP2 |
| PCR_NSP2_R | GGCTTTTTAAAGCGTCTCAGT | - | Amplification of NSP2 |
| PCR_NSP4_F | AGTTCTGTTCCGAGAGACC | - | Amplification of NSP4 |
| PCR_NSP4_R | GGWYACRYTAAGACCRTTCC | - | Amplification of NSP4 |

*Bold letters show silent mutations introduced to engineer restriction enzyme recognition sites.

#Grey shading indicates restriction enzyme sites.

**Supplementary Results**

**Supplementary Figure 1: Rescue of rSA11-mNSP4. A**) Extracted dsRNA for rSA11 and rSA11-mNSP4. **B**) Restriction enzyme digestion analysis of amplified genome segments 8 (NSP2) and 10 (NSP4). A 2.0 % agarose gel indicating the restriction enzyme digestion. Lane 1 shows an uncut genome segment 8 (wtSA11) and lanes 2-3 shows the respective mutant genome segment 8 cut with BstUl resulting in two products – 800bp and 150bp. Lane 5 shows an uncut genome segment 10 (wtSA11) and lanes 6-7 shows the respective mutant genome segment 10 cut with Xbal resulting in two products – 638bp and 100 bp (indicated by arrow). **C**) Protein alignment of rSA11 and rSA11-mNSP4 following Illumina MiSeq sequencing of rescued viruses. Changes in the viroporin domain (75-80) of rSA11-mNSP4 is indicated by a red box.
